# *Pseudomonas aeruginosa*: An Audacious Pathogen with an Adaptable Arsenal of Virulence Factors

**DOI:** 10.3390/ijms22063128

**Published:** 2021-03-18

**Authors:** Irene Jurado-Martín, Maite Sainz-Mejías, Siobhán McClean

**Affiliations:** School of Biomolecular and Biomedical Sciences, University College Dublin, Belfield, Dublin 4 D04 V1W8, Ireland; irene.juradomartin@ucd.ie (I.J.-M.); maite.sainzmejias@ucd.ie (M.S.-M.)

**Keywords:** *Pseudomonas aeruginosa*, virulence factors, adaptation, cystic fibrosis, diversity, genomics, lung environment

## Abstract

*Pseudomonas aeruginosa* is a dominant pathogen in people with cystic fibrosis (CF) contributing to morbidity and mortality. Its tremendous ability to adapt greatly facilitates its capacity to cause chronic infections. The adaptability and flexibility of the pathogen are afforded by the extensive number of virulence factors it has at its disposal, providing *P. aeruginosa* with the facility to tailor its response against the different stressors in the environment. A deep understanding of these virulence mechanisms is crucial for the design of therapeutic strategies and vaccines against this multi-resistant pathogen. Therefore, this review describes the main virulence factors of *P. aeruginosa* and the adaptations it undergoes to persist in hostile environments such as the CF respiratory tract. The very large *P. aeruginosa* genome (5 to 7 MB) contributes considerably to its adaptive capacity; consequently, genomic studies have provided significant insights into elucidating *P. aeruginosa* evolution and its interactions with the host throughout the course of infection.

## 1. Introduction

*Pseudomonas aeruginosa* is a significant cause of healthcare-associated infections, being particularly problematic in intensive care units. Its infections are associated with high morbidity and mortality in many groups, including individuals with healthcare-associated pneumonia, chronic obstructive pulmonary disease (COPD), or cystic fibrosis (CF) [1,2,3,4,5,6,7,8]. It is included in the “critical” category of the World Health Organisation’s (WHO) priority list of bacterial pathogens for which research and development of new antibiotics are urgently needed [9,10].

As a versatile opportunistic pathogen, *P. aeruginosa* is capable of causing both acute and chronic infections. Its pathogenic profile stems from the large and variable arsenal of virulence factors and antibiotic resistance determinants harboured in *P. aeruginosa*’s genome, which confer remarkable metabolic flexibility and the ability to adapt to multiple conditions, including the host immune response [1,11,12,13]. *P. aeruginosa*–host interactions are still poorly understood, complicating the development of effective therapies and vaccines. There are still no vaccines available to prevent these infections despite a half century of research effort specifically focussed on this challenge, as recently reviewed [14]. While CF patients are colonised by both *P. aeruginosa* and *Staphylococcus aureus* during their childhood, in adulthood, *P. aeruginosa* predominates, contributing to lung function decline [15,16]. The reasons for *P. aeruginosa* persistence in CF airway are multifactorial, and the relationship between pathogen traits and host factors that enables the development of chronic infections is highly complex [12]. Nevertheless, it is widely known that the CF environment confers multiple advantages on *P. aeruginosa* enabling its colonisation of CF airways over other pathogens, such as *S. aureus* and *Klebsiella pneumoniae* [15,17]. Consequently, the prevalence of *P. aeruginosa* in adults with CF ranges from 31% (in Ireland) to 47% (in the US) in recent studies [18].

The genetic and phenotypic properties of persistent *P. aeruginosa* strains in CF airways differ greatly from those that initiated the infections [19,20], as *P. aeruginosa* undergoes evolutionary changes in response to the selective forces in CF airways [5]. Understanding the mechanisms of the *P. aeruginosa* adaptation and evolution during chronic respiratory CF infections could be key to finding of novel therapies against *P. aeruginosa* infections. This review summarises the multiple virulence factors that provide *P. aeruginosa* its metabolic flexibility and describes the arsenal of tools that allow *P. aeruginosa* to persist in the hostile CF environment, highlighting the adaptations by *P. aeruginosa* throughout the different stages of the infection. The large *P. aeruginosa* genome (~5 MB to ~7 MB), which comprises multiple genetic regulatory pathways, is also key to understanding the pathoadaptability of this pathogen, especially with current genomic techniques which allow the assessment of differences and similarities across *P. aeruginosa* populations colonising CF airways.

## 2. *Pseudomonas aeruginosa* Virulence Factors: A Wealth of Weaponry

**Figure 1:**
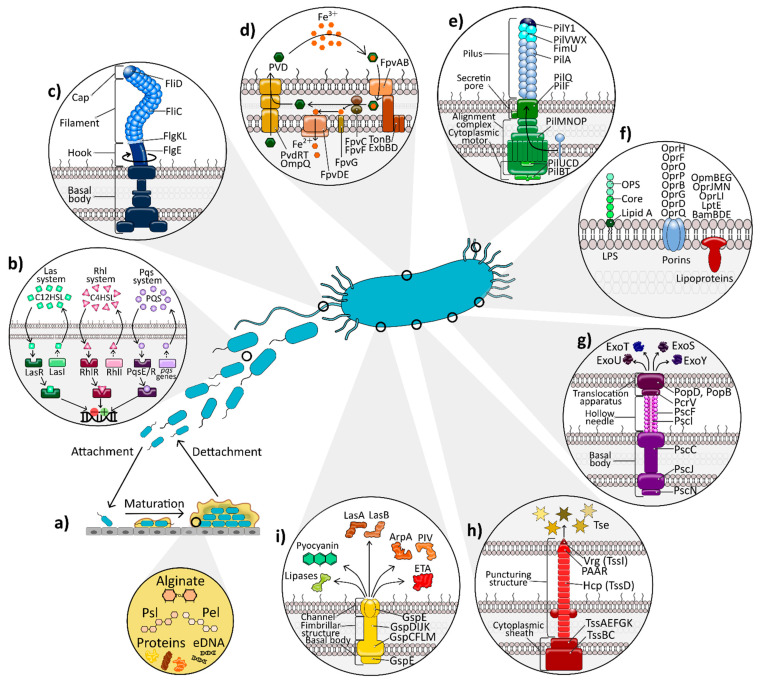
Schematic presentation of the main virulence factors used by *P. aeruginosa* during respiratory infections: (**a**) biofilm formation ability and composition of the extracellular matrix of biofilms (exopolysaccharides, proteins and extracellular DNA); (**b**) the three main quorum sensing (QS) systems (Las, Rhl and Pqs); (**c**) flagellins FliC and FliD incorporated within the flagellar structure; (**d**) pyoverdine (PVD) siderophore as an iron uptake system; (**e**) type 4 pili (T4P); (**f**) lipopolysaccharide (LPS) and outer membrane proteins (OMPs); (**g**) the type III secretion system (T3SS) and its four main effectors; (**h**) the type VI secretion system (T6SS); (**i**) the type II secretion system (T2SS) and the compounds it releases to the extracellular milieu: lytic enzymes (lipases, proteases (AprA ad PIV) and elastases (LasA and LasB)), exotoxin A (ETA), and pyocyanin.

*P. aeruginosa* displays a vast repertoire of both cell-associated and extracellular virulence factors that contribute to its pathogenesis, being controlled by incredibly complex, interconnected regulatory circuits and signalling systems, which give this pathogen great plasticity [21,22]. Here, we review the structure and function of the most relevant virulence factors in respiratory infections (Figure 1).

### 2.1. The Outer Membrane: Lipopolysaccharide and Proteins

The outer membrane (OM) of *P. aeruginosa* has an asymmetric bilayer that limits the entry of harmful compounds, with a phospholipid inner face and a lipopolysaccharide (LPS) outer face, embedded with about 300 proteins (OMPs) that play different roles, most of which remain unknown (Figure 1) [23,24,25,26].

#### 2.1.1. Lipopolysaccharide

Lipopolysaccharide is comprised of three domains: the lipid A, the core region, and the O-antigen or O-polysaccharide (OPS) [27], and various glycoforms which all contribute to its virulence are produced (Section 4) [28]. It constitutes a physical barrier, mediates interactions with host receptors, and causes tissue damage due to its endotoxic activity [29]. LPS stimulates the production of reactive oxygen species (ROS) and gel-forming mucin in airway epithelial cells, which is associated with morbidity and mortality of patients with asthma, COPD, and CF [30,31]. It increases airway epithelial paracellular permeability [32] and induces pulmonary inflammation by stimulating tumour necrosis factor-α (TNF-α), Interleukin (IL)-1, IL-6 and Interferon (IFN)-γ [33]. LPS also contributes to antibiotic resistance and influences the formation of outer membrane vesicles (OMVs) and biofilms [28].

Lipid A is a hydrophobic glycolipid that anchors the other two moieties of LPS into the OM and mediates endotoxicity. It is composed of a diglucosamine biphosphate backbone with O- and N-linked fatty acids and varies among isolates depending on the growth conditions and isolation sources, which has considerable implications for niche adaptation (Section 4) [34]. The acyl chains of the lipid A bind to host cell MD2 receptor, activating the toll-like receptor (TLR)-4 signalling pathway [35]. Both the acyl chains and the phosphates of the lipid A interact with a quinolone of the Pqs quorum sensing (QS) system when it is exported to the OM, inducing membrane curvature, leading to OMV formation [36]. Mutants defective for lipid A synthesis failed to develop biofilms on both biotic and abiotic surfaces and exhibited significantly decreased bacterial attachment to airway epithelial cells, suggesting that LPS may play an indirect role in bacterial adhesion and biofilm formation [37].

O-polysaccharide is a highly variable and immunogenic peripheral long chain of repeating polysaccharides that can be either linear or branched [27]. Two forms of LPS are exposed on bacterial surface, called “capped” or “smooth” and “uncapped” or “rough” when the OPS is present or absent, respectively [34]. Additionally, two O-antigens are simultaneously produced: (i) the common polysaccharide antigen (CPA or A-band), a homopolymer that has a conserved structure consisting of repeating units of d-rhamnose trisaccharide and (ii) the O-specific antigen (OSA or B-band), a strain-variable heteropolymer that gives rise to 20 serotypes, according to scheme proposed by the International Antigenic Typing System (IATS) [34]. Because OPS extends outward from the OM, it is involved in many host–pathogen interactions: (a) the prevention of bacterial killing by inhibiting the deposition of pore-forming membrane attack complex and phagocytosis [28], (b) the protection from oxidative stress [29], and (c) likely, NETosis stimulation [38]. Changes in the OPS affect OMV size and protein content [39], although the mechanism is still poorly understood. Additionally, cells that do not produce CPA failed to develop into robust biofilms and exhibited changes in cell morphology and biofilm matrix production [39], probably because OPS is essential for effective motility [40]. CPA may also be important for bacterial attachment to human bronchial epithelial cells [34,41].

#### 2.1.2. Outer Membrane Proteins

##### Porins: OprF, OprH, and OprD Superfamily

*P. aeruginosa* expresses 26 specific β-barrel channel proteins for the exchange of different molecules, although most of them are multi-functional [42] (Table 1). The major porin OprF is the most abundant non-lipoprotein OMP and belongs to the OmpA family. It has three domains: (i) a N-terminal eight-stranded β-barrel located in the OM, (ii) a partly surface-exposed cysteine-rich linker, and (iii) the C-terminal region with α-helixes and/or β-strands with a peptidoglycan (PG) binding domain [42]. OprF is essential for *P. aeruginosa,* for both OM integrity and virulence. It has open and closed conformations, with the closed form representing more than 95% of the OprF population, contributing to the extremely low permeability of the OM [43] and to the maintenance of OM integrity and cell shape under low osmolarity conditions by binding to the PG and other OMPs (OprL and OprI) [42,44]. It is responsible for the acquisition of non-specific ions and saccharides [26] and may allow the diffusion of toluene, siderophores, nitrates, and nitrites [42]. OprF also mediates bacterial adhesion to human alveolar epithelial cells and to other bacteria to form microcolonies, probably via an OprF-lectin B complex [42,43]. OprF mutants show a reduced ability to attach to other cells and to produce virulence factors, such as pyocyanin (PYC), elastase (LasB), lectin PA-1L, and exotoxin A (ETA) [45,46]. Since it is particularly abundant in the OM of sessile cells [42], OprF may also influence biofilm development, but conflicting results have been obtained. For example, an OprF mutant showed a decrease in biofilm formation [46], and OprF has found to be indispensable for growth in anaerobic biofilms [47] and for sensing surfaces during the attachment stage of biofilm formation [48]. In contrast, another study reported that the absence of OprF was correlated with an increase in biofilm formation due to the upregulation of c-di-GMP [49]. OprF also modulates the expression of the type III secretion system (T3SS) and its effectors during both the extracellular and intramacrophage lifestyle [45,50]. It has also been suggested to act as a sensor of the host immune system as a C3b receptor [51] and binding to the IFN-γ [43], modulating QS systems to trigger a virulence response [43,47]. Moreover, it offers protections from macrophage clearance in acute infections [52].

OprH, a member of the OmpW family and the second smallest *P. aeruginosa* porin, binds to the surfactant protein A (SP-A) [53] and laminin during respiratory tract infections [54]. It is involved in antibiotic resistance to aminoglycosides and polymyxins [42], and may contribute to OM integrity by interacting with the LPS [58,59]. It may be involved in transportation of hydrophobic molecules, cations, iron (Fe^2+^), and small aminoacids via conformational changes [42,50].

The OprD (Occ) superfamily includes 19 members that share a high degree of similarity but differ in substrate specificities. They are split into two subfamilies: OccD (involved in basic amino acids uptake) and OccK (for the negatively charged cyclic molecules). Among them, OprD (OccD1) is the second major porin protein, involved in the entry of carbapenem antibiotics and transportation of basic amino acids, peptides, and probably gluconate [42]. It showed the greatest overrepresentation in OMVs from biofilm samples, displays protease activity, and, similar to OprG, it binds to laminin [42,54]. OprQ (OccD6) is also important for adhesion to epithelial cells by binding to human fibronectin [55].

##### Lipoproteins

Lipoproteins can be grouped into different families according to their function (Table 1). Some are part of the assembly machinery used for OM biogenesis, such as BamBDE or LptE [24]. Others participate in the maintenance of the cell integrity through interacting with PG, including OprL (the most abundant lipoprotein) and the small OprI [24]. OprL also contributes to protecting cells against oxidative stress [56]. Finally, OprM, OprN, OprJ, OpmG, OpmB, or OpmE are involved in the efflux of harmful molecules, including antibiotic drugs, thus conferring antibiotic resistance [24,57].

### 2.2. Biofilm Formation

*P. aeruginosa* is renowned for developing robust biofilms that are highly resistant to antibiotics, disinfectants, and host defences [60,61], impairing bacterial clearance and leading to the establishment of highly recalcitrant chronic infections that are a major medical issue [60]. More than 50% of ECM of *P. aeruginosa* is formed by three exopolysaccharides (EPSs): the capsular polysaccharide alginate and two aggregative polysaccharides (Psl and Pel), but it also contains extracellular DNA (eDNA) and proteins [62] (Figure 1a). Mature *P. aeruginosa* biofilms are hallmarked by “capped” mushroom-shaped structures and a complex network of channels that distribute nutrients and oxygen and remove waste products [61,62].

Biofilm development is multifactorial. Initiation occurs with an increase in c-di-GMP, an intracellular second messenger that induces adhesins and EPS biosynthesis and physiological changes necessary to switch from motile planktonic growth to a sessile biofilm-associated lifestyle [63]. The secretion of various QS-controlled extracellular enzymes (esterases, lipases, and elastases) affects EPS composition, the properties of the ECM, and cell motility, thereby influencing formation and architecture of mucoid *P. aeruginosa* biofilms [64,65,66,67]. Recently, it has been hypothesised that QS may also control the dispersal step [61]; in fact, quinolone-induced OMV formation may facilitate cell dispersal [68]. Finally, small RNAs also regulate biofilm formation [69]. Of note, a recent study showed that there are several pathways to develop biofilms and that expression of genes regulating stress responses and adaptation to oxygen and iron-limited environments is vital for this process [70].

#### Alginate

Alginate, also referred to as mucoid exopolysaccharide (MEP), is the most studied EPS and the major component of mucoid *P. aeruginosa* biofilms. It is a high molecular weight, random polymer with variable ratios of D-mannuronic and L-guluronic acids that are β1-4 linked and partially O-acetylated. The *algD* operon encodes the enzymes required for alginate synthesis, and its expression is regulated by AlgT σ-factor [62], whose overexpression is lethal to mucoid strains [71]. This exopolysaccharide is overproduced by mucoid *P. aeruginosa* strains, and despite not being a requirement for biofilm formation [72,73], it helps its maturation, architecture, and stability [73,74]. Alginate attaches to tracheobronchial mucin, serving as alternative adhesin [75]. The acetyl groups contribute to its highly viscosity, allowing the retention of water and nutrients [62]. Importantly, alginate contributes to bacterial persistence by protecting *P. aeruginosa* against host phagocytosis in the lungs [76] and scavenging ROS released by activated macrophages and neutrophils [62]. In addition, alginate and Psl elicit a strong polymorphonuclear leukocyte response, leading to a substantial release of ROS that contributes to lung inflammation [77]. Finally, it may bind aminoglycoside antibiotics, such as tobramycin, impairing their penetration into the biofilm and enhancing antibiotic resistance [78].

### 2.3. Flagellum

*P. aeruginosa* possesses a single polar flagellum that consists of a filament made of helicoidally arranged, polymerised flagellin (FliC), a type-specific cap protein (FliD), the hook at the base of the filament (FlgE), two filament-hook junction proteins (FlgKL), and a number of basal body components across outer and inner membranes [79,80] (Figure 1c). FliC protein consists of three domains: D0, D1 (a conserved and immunogenic structure) and D2 (a variable domain) [81], and is divided into two serotypes: the heterogeneous Fli-a and the serologically uniform Fli-b.

Although *P. aeruginosa* also swarms over solid surfaces, the flagellum is primarily responsible for swimming motility in aqueous or low viscosity environments through rotation in a corkscrew manner, generating a force that moves the bacterium forward [80]. Chemotaxis is important for *P. aeruginosa* initial binding to the CF airway epithelia since it is used to direct flagella-mediated swimming towards these cells [82]; consequently, the flagellum is considered an important virulence factor [83]. Aside from motility, FliC protein is responsible for binding to (i) the membrane glycolipid asialo-GM1 on the apical surface of the lung epithelial cells [79], (ii) heparan sulphate proteoglycans localised on the basolateral surface [84], and (iii) SP-A surfactant [85], whereas FliD mediates adhesion to human respiratory MUC1 mucin [79]. Furthermore, PAO1 mutants lacking flagellar proteins such as FlgE (flagellar hook protein) lost their resistance to SP-A surfactant protein, showing that the flagellum is involved in pathogenesis beyond motility [86]. These interactions all trigger the TLR5 pathway [79]. Flagellar attachment ability aids initial biofilm establishment, while motility allows cell dispersal in the final steps; however, an adequate timing of motility control is required to form robust biofilms during the maturation stage [87].

### 2.4. Type IV Pili

Type IV pili are retractable, hair-like filamentous appendages which are polarly located. They are composed of thousands of molecules of a small monomeric protein, major pilin (PilA), along with less abundant minor pilins localised at the tip of the pilus (FimU-PilVWXY1), which are subdivided into core minor pilins (important for pili formation and tip stabilisation) and non-core minor pilins (involved in aggregation, adhesion and DNA uptake) [88]. The function of the pili is accomplished through a powerful machinery organised in (i) the cytoplasmic motor subcomplex (PilBTUCD), (ii) the inner membrane (IM) alignment subcomplex (PilMNOP), and (iii) the OM secretin pore subcomplex (PilQF) [88] (Figure 1e). Finally, PilA is formed by three domains: (i) a highly conserved hydrophobic N-terminal α-helix region, (ii) a hypervariable central region, and (iii) a semi-conserved C-terminal region containing the binding domain to host epithelial cells through a disulfide-bound loop region [88]. Additionally, there are five groups of T4P (I, II, III, IV, and V) that are associated with different patterns of biofilm production and multidrug resistance (MDR) [89].

Pili are essential structures for the initiation of the infection by mediating motility and adhesion. They control twitching motility, used for rapid colonisation of different surfaces [90]. This involves sequential cycles of extension, adhesion, and retraction of T4P fibres, which generate the force to drive the cell forward [91]. Extension and retraction of the pilus is achieved through two cytoplasmic membrane-associated ATPases (PilB and PilT) which, respectively, polymerise and depolymerise PilA subunits at their base [90]. The minor tip pilin PilY1 specifically recognises a host receptor localised on the basolateral surface of epithelial cells, and binds integrin in RGD- (arginine–glycine–aspartic acid) in a calcium-dependent manner [92,93]. PilY1 is also required for the expression and stabilisation of T4P [92] and is thought to be a mechanosensor for *P. aeruginosa* attachment-induced virulence, along with other minor pilins [94,95]. Finally, pili may also bind to the glycolipids asialo-GM1 and asialo-GM2, a tip-associated interaction mediated by the C-terminal region of the pilin A [96,97]. However, some studies indicate that various *P. aeruginosa* clinical isolates do not employ such gangliosides during the attachment event [98]. Due to their adhesive and motility properties, T4P also play a role in biofilm development and aggregation and the formation of mushroom-like biofilm cap structures [99]. Moreover, the pilus tip can bind DNA, which is likely involved in natural transformation and biofilm formation [100]. Finally, it confers resistance to SP-A-mediated phagocytosis [101] and activates the inflammasome [102].

### 2.5. Protein Secretion Systems

*P. aeruginosa* possesses five secretory systems that secrete a wide variety of toxins and hydrolytic enzymes to attack the host [103,104]. The type I and V secretion system (T1SS and T5SS) are the simplest secretion pathways and release products to the extracellular milieu. T1SS releases the alkaline protease AprA, the haemophore HasAp, the AprX with unknown function, and TesG, which supresses neutrophil influx during chronic infections [103,105]. T5SS secretes EstA esterase, CdrA extracellular adhesin, and LepA exoprotease [103]. The type II secretion system (T2SS), or secreton, is the most versatile system used by *P. aeruginosa,* releasing a broad diversity of exoproteins (Section 2.6), being composed of at least 10 proteins that are divided into the basal body in the IM, the fimbrillar structure in the periplasmic space, and the channel that crosses the OM (Figure 1i) [103]. The most important secretion system is the T3SS, used for the disabling and destruction of the host’s immune system [106]. Most recently discovered, the type VI secretion system (T6SS) is made of a Hcp (TssD) tube with two spike proteins that is propelled by a cytoplasmic sheath (Figure 1h). It is important for bacterial competition, as it produces bacterial toxins (Tse) that destroy host microbial flora, although it also plays a minor role against host defences [107]. While T2SS and T5SS use a two-step secretion mechanism, involving a stopover of the secreted proteins into the periplasm, the other three systems use a one-step mechanism. Both T3SS and T6SS mediate virulence by directly injecting exoproteins into the cytoplasm of the targeted cell [103,104].

#### 2.5.1. Type III Secretion System

The *P. aeruginosa* T3SS injects toxic effectors directly into the host cell cytosol. It is a syringe-like “injectisome”, comprised of at least 20 proteins, and divided into (i) the secretion apparatus, which transports effectors through the bacterial membranes and (ii) the translocation apparatus, which translocate the effectors through host cell membrane [106] (Figure 1g). The secretion apparatus possesses a hollow needle of a helically polymerised protein (PscF) and a basal body, comprised of a cytoplasmic ATPase (PscN), an IM lipoprotein ring (PscJ), and an oligomerised secretin ring at the OM (PscC). The translocation apparatus is made of two hydrophobic proteins (PopB and PopD), which interact with the host cell membrane to form the translocation pore, and a hydrophilic protein PcrV, essential for correct assembly and insertion of PopB and PopD into host cell membranes [106,108]. The injectisome is expressed at basal levels, existing in a quiescent state until various inducing signals (low concentrations of extracellular Ca^2+^, serum albumin/casein, and host cell contact via pilins or flagella) promote its expression, controlled by the master regulator ExsA [109]. Although not required for infection, T3SS enhances disease severity. It enables *P. aeruginosa* to cause epithelial injury, disseminate into the circulation, and counteract host innate immune responses in an effector-independent or effector-dependent way [106].

##### Effector-Dependent Pathogenicity

There are four well-known toxic effectors injected via T3SS that are variably expressed in different strains: ExoU, ExoT, ExoS, and ExoY [106]. Two novel effectors have been proposed (PemA and PemB) [110], and other proteins may also be translocated through this system, such as flagellar proteins [111], PilA [102], and nuclear diphosphate kinase [112].

Despite being produced by less than half of clinical isolates (24–42%) [113], ExoU is considered the major T3SS cytotoxin because it has the greatest impact on disease severity, being associated with severe acute lung injury, sepsis, and mortality [114,115,116]. It has a phospholipase A2 activity that irreversibly destroys the host cell membrane, causing rapid cell death. This killing activity may be directed against phagocytes and the epithelium to promote bacterial dissemination and prevent clearance [117]. ExoU is also associated with a proinflammatory response by augmenting eicosanoid production in both epithelial cells and neutrophils. It activates NF-κB, stimulating IL-8 secretion during the infection process and leading to increased neutrophil recruitment across the infected pulmonary epithelium [118,119].

ExoY is the second most prevalent exotoxin, expressed by >89% of isolates [113,120]. It is a soluble adenylate cyclase that elevates the intracellular levels of various cyclic nucleotides (cAMP, cCMP, cGMP, and cUMP) when injected into mammalian cells, activating protein kinases [117,121]. Consequently, it causes irreversible actin microtubule disassembly, cell necrosis, and alteration of endothelial barrier integrity, following lung injury and end-organ dysfunction [120,122]. Recently, it was shown that ExoY possesses one actin-binding site that directly bundles actin filaments in the host cell [123]. Strikingly, ExoY downregulates the activation of transforming growth factor β-activated kinase 1 (TAK1), thereby inhibiting the production of proinflammatory cytokines by both macrophages and epithelial cells [124].

ExoT is the most prevalent exotoxin produced by clinical isolates (92–100%) [113], although it is not sufficient for bacterial persistence in the lung on its own [114]. It is a bifunctional exotoxin with GTPase activating protein (GAP) and adenosine diphosphate ribosyl transferase (ADPRT) activities, which work synergistically to impede phagocytosis and disrupt epithelial barriers [117]. The GAP domain inactivates three small GTPases (Rac, Rho, and Cdc42) that maintain the organisation of the host cell cytoskeleton, leading to a reversible disruption of the actin cytoskeleton that inhibits cell migration and induces cell rounding and detachment. Inactivation of Rho also involves cytokinesis inhibition [117]. Moreover, the GAP activity of ExoT, along with ExoS, contributes to feedback inhibition of effector injection [125]. ExoT specifically ribosylates two adaptor proteins (CT10-regulator of kinase (Crk)-I and Crk-II) that play a role in phagocytosis, focal adhesion, and cell migration [126]. ExoT also increases IFN-γ production by natural killer cells in the lung [127].

ExoS, whose production was recently associated with chronic infections and worse clinical outcomes in CF patients [128] is found in 58–72% of the clinical isolates [113] and possesses the same bifunctional activity as ExoT [117]. Its GAP activity is directed towards the same three GTPases, but unlike ExoT, the ADPRT domain of ExoS targets a wide range of cell factors and pathways, thereby producing several adverse effects on the host cells, such as cell death, actin cytoskeletal disruption, inhibition of DNA synthesis, vesicular trafficking, or endocytosis [117]. Early during the infection, ExoS is mainly injected into neutrophils, where the ADPRT activity is the main contributor in preventing phagocytosis [129,130]. In fact, ADP-ribosylation of Ras protein blocks ROS production in these cells [131]. Later in the infection, type I pneumocytes are injected with ExoS, resulting in pulmonary-vascular barrier disruption [129]. ExoS also ribosylates and inactivates the ezrin, radixin, and moesin (ERM) family of proteins, involved in motility, phagocytosis, adhesion, and cell shape maintenance [117]. Finally, ExoS activates TLR2 and TLR4 pathways [132].

##### Effector-Independent Pathogenicity

While most of the damage is mediated by its effectors, the T3SS itself also contributes to pathogenicity. One study showed that PopB contributed to mortality in the absence of any of the effectors, and that the translocation channel triggers the activation of IL-1β and prevents bacterial clearance [133]. Although it remains controversial, *P. aeruginosa* likely exploits the activation of a host innate immune cytosolic sensor, the inflammasome, to the detriment of the host itself [111,134]. Its activation via inner-rod PscI and needle PscF proteins leads to the secretion of IL-18, which is involved in two processes: (i) the downregulation of IL-17 production in the lung, dampening the production of lung epithelial antimicrobial peptides (AMPs) that eliminate bacteria and (ii) excessive neutrophil recruitment, resulting in lung injury [134].

### 2.6. Other Released Products

#### 2.6.1. Exotoxin A

The most toxic *P. aeruginosa* virulence factor is the exotoxin A, an ADP-ribosyl transferase secreted through the T2SS by the majority of clinical isolates [113]. It is subdivided into three structural prominent domains and one minor subdomain. The N-terminal domain (Ia) is composed primarily of antiparallel β-strands and is responsible for attachment to host cells; the middle domain (II), composed of six α-helices with membrane translocating activity; and the and C-terminal domain (III) is the toxic moiety. There is a minor Ib subdomain, located between domains II and III, that can be removed without loss of toxin activity [135].

When ETA is released to the extracellular surroundings, it binds to host cells through CD91 or α2-macroglobuline receptor, leading to its internalisation via clathrin-coated pits or detergent-resistant microdomains. Once inside, it undergoes conformational changes that enable its irreversible necrotising activity at the site of colonisation [135]. Due to its ADP-ribosylating activity, it inhibits host protein synthesis by inactivating the eukaryotic elongation factor 2 (eEF-2), a member of the GTPase superfamily that translocates the mRNA within ribosomal sites [135]. ETA also provokes the activation of two caspases involved in the apoptosis process [136,137], inhibits IL-18 secretion, and decreases TNF-α, IL-6, IL-8, and IL-10 production [33,138,139].

#### 2.6.2. Proteolytic Enzymes

*P. aeruginosa* releases a wide range of extracellular proteases that are critical for invasion in acute infections: LasA and LasB elastases, alkaline protease (AprA), type IV protease (PIV), *P. aeruginosa* small protease (PASP), Large ExoProtease A (LepA), *P. aeruginosa* aminopeptidase (PAAP), and MucD [75,140].

LasA and LasB elastases are secreted by the T2SS under the regulation of QS systems [141] and degrade host elastin [75,142]. LasB elastase, also named “elastase” or “pseudolysin”, is a zinc-dependent metalloprotease of the thermolysin family encoded by *lasB* gene. It is the most abundant protease and is considered the principal extracellular virulence factor [140]. Apart from its elastinolytic activity, it also disrupts epithelial tight-junctions [143] and cleaves other host proteins, for instance, surfactant proteins (SP-A and SP-D) [144,145], cytokines (TNF-α, IFN-γ, IL-6 or IL-2), immunoglobulins [140,145,146], and components of the inflammasome [147], thereby interfering with bacterial clearance. It also degrades exogenous flagellin under calcium-replete conditions, avoiding TLR5 recognition [148] and undermines alveolar macrophage activity through downregulation of the production of important secreted ROS and innate immune molecules and receptors [149]. It also affects biofilm formation through rhamnolipid (RL) regulation [150]. LasA, or staphylolysin, is a serine protease encoded by *lasA* gene, its name owing to its ability to cause rapid lysis of *S. aureus* by cleaving the pentaglycine bridge in its PG [140]. Although its elastinolytic activity is limited, it can enhance this action in other proteases, including LasB, by cleaving the glycine–glycine bonds within elastin [75,140]. Recently, LasA expression was correlated with antibiotic resistance in *P. aeruginosa* clinical isolates [151].

Alkaline protease, or aeruginolysin, is a zinc-dependent metalloendopeptidase secreted through the T1SS and encoded by *aprA* gene [140,142]. It mainly interferes with fibronectin and laminin, two components of the endothelium [140], and degrades complement proteins (C1q, C2, and C3) and cytokines (IFN-c, TNF-a and IL-6) [152], allowing for phagocytic evasion. It also cleaves free flagellin monomers [153] and may reduce mucocilliary clearance of bacteria by activating the epithelial sodium channel (ENaC) [154]. It also contributes to the production of other virulence factors, such as PYC [155].

This Type IV protease is a serine protease secreted by T2SS that belongs to the chymotrypsin family S1 [140]. It is encoded by the *piv* gene and its expression is under the control of the Las QS system [140,156]. Although it has a major role in corneal virulence, it may also be important to *P. aeruginosa* pathogenesis within the CF lung by degrading the fibrinogen and the surfactant proteins (SP-A, SP-D and SP-B), contributing to tissue invasion and damage [140,157,158]. PIV also promotes immune evasion by degrading plasminogen, immunoglobulin, C1q, C3, and IL-22, which hampers mucosal defence regulation and exacerbates pneumococcal pneumonia and invasive disease [140,159,160]. It may also interfere with the activation of the Toll signalling and the production of (AMPs) [161].

#### 2.6.3. Lipolytic Enzymes

Lipases are secreted by the T2SS and cleave lipids, yielding free fatty acids and glycerol as final products [103]. LipA is the major lipase produced by *P. aeruginosa* and needs the chaperon *lif* to be activated [162]. It is encoded in the *lipA/lipH* operon, along with its cognate foldase LipH, which is also required for the expression of LipC [64]. These enzymes may be expressed in response to variable environmental conditions [163] and affect other virulence factors [64,65,66,67]. Recently, a new lipase (A12) was identified [164].

EstA esterase hydrolyses glycerol esters with short- or long-chain fatty acid and is auto-transported through T5SS, locating in the OM [103]. One study showed its high affinity-binding to laminin during respiratory tract infections, displaying an adhesion function owing to this location [54]. Similar to LipA and LipC, it is crucial for the functioning of other virulence factors, i.e., RL production, cell motility, and biofilm formation [66].

Phospholipase C is secreted through T2SS and breaks down eukaryotic membrane phospholipids and sphingomyelin, also possessing haemolytic activity [165]. Its production compromises lung function by causing dysfunction of mammalian pulmonary surfactant [166] and modulates IL-8 production in the lung of CF patients [167].

#### 2.6.4. Pyocyanin

Pyocyanin is a redox-active secondary metabolite responsible for the blue-greenish colour of *P. aeruginosa* colonies in culture [142]. This phenazine, secreted by the T2SS, is associated with disease severity and lung function decline due to its free radical and pro-inflammatory effects [168]. It can increase intracellular ROS and H_2_O_2_, provoking oxidative stress and damaging components of the cell cycle, several enzymes, and DNA, leading to cell lysis [168]. Consequently, eDNA is released, likely contributing to biofilm formation and helping in the persistence of infections [169]. Mitochondrial ROS release is also induced, leading to neutrophil apoptosis [170]. Additionally, it slows ciliary beating, causes epithelial disruption, and increases mucous secretion in the respiratory tract, contributing to lung colonisation. It also increases IL-8 production by alveolar macrophages and neutrophil influx [168].

### 2.7. Other Bacterial Products

#### 2.7.1. Rhamnolipids

Rhamnolipids are amphipathic, extracellular, secondary metabolites formed by a mono- or di-(L)-rhamnose moiety (hydrophilic group) linked via an O-glycosidic bond to a dimer of β-hydroxy fatty acid tail (hydrophobic group). They contribute significantly to *P. aeruginosa* pathogenesis in the lung, by degrading lung surfactant [171], reducing transepithelial electrical resistance [172], and disrupting tight junctions [173] in the respiratory epithelium. Their production by colonising isolates has been associated with ventilator-associated pneumonia (VAP) development [174]. While overproduction of RLs impedes biofilm development, low concentrations enhance LPS release to the cell surface, increasing hydrophobicity and affinity for initial adherence of bacteria to a surface [175,176]. The production of an appropriate amount contributes to biofilm architecture by maintaining open the non-colonised channels [177]. RLs also facilitate sliding motility in the absence of T4P and flagella [178] and allow swarming motility by lowering the surface tension due to its surfactant properties [179,180]. Moreover, RL production is induced under iron-restricted conditions, promoting twitching motility [181]. They also participate in immune evasion to facilitate chronic infections by causing necrotic killing of polymorphonuclear leukocytes [182,183]. They suppress the host innate immunity, preventing a flagellin-induced human β-defensin 2 response through targeting protein kinase C [184].

#### 2.7.2. Antioxidant Enzymes

*P. aeruginosa* expresses a number of antioxidant enzymes that help it overcome oxidative stress in the host, including three catalases (KatA, KatB, and KatC or KatE), four alkyl hydroperoxide reductases (AhpA, AhpB, AhpCF, and Ohr), and two superoxide dismutases (SodA and SodB) [185]. KatA and KatB catalases protect planktonic and sessile cells against H_2_O_2_ and other free radicals produced using H_2_O_2_ as a substrate [185]. Their expression and activity are regulated through different pathways (OxyR, IscR, RpoS, and the stringent response) [186,187,188] and also by QS in the case of the major catalase KatA [185]. KatA is constitutively expressed at high levels and is present in either the cytoplasm or the periplasm, suggesting that it could be a released protein, maybe through cell lysis [185,189]. Its versatile catalase activity allows for the detoxification of reactive nitric species (RNS) under anaerobic conditions, peroxide resistance, and osmoprotection [190,191]. By contrast, KatB is inducibly expressed upon exposure to H_2_O_2_ and is localised only within the cytoplasm, having an auxiliary function to assist KatA under oxidative stress conditions [185,190].

### 2.8. Iron Acquisition Systems

Iron (Fe^3+^) is an essential nutrient for bacterial growth and virulence. In the stressful host environment, iron is not readily available due to its low solubility and the activity of host iron-binding proteins (transferrin and lactoferrin) [192]. To fulfil iron requirements, *P. aeruginosa* uses different strategies: (i) production of low-molecular weight, organic compounds called siderophores (pyoverdine and pyochelin); (ii) xenosiderophores uptake; (iii) haeme molecule uptake from the host haemoproteins via two systems (Has and Phu); and (iv) iron reduction by phenazines through the Feo system [193].

#### Siderophores: Pyoverdine and Pyochelin

While pyochelin is a salicylate-based siderophore with a lower affinity for iron, pyoverdine (PVD) has a peptide nature and is considered the major siderophore [192]. Since PVD production is an energy-demanding process, *P. aeruginosa* primarily produces pyochelin, and only when iron concentration becomes really low, it switches to PVD production [193]. PVD is comprised of a variable peptide chain and a conserved dihydroxyquinoline chromophore, which binds iron. More than 50 pyoverdines are produced by different strains, although they can be grouped into three types (PVDI, PVDII, and PVDIII) based on differences in the peptide chain [192]. PVD both chelates free iron and scavenges it from host proteins, and this is accomplished by a complex network of membrane and periplasmic efflux-pumps and transporters [194] (Figure 1d). Despite being essential, iron catalyses Fenton reactions producing ROS at high concentrations, leading to cytotoxicity [195]. Thus, this system is shut off in the presence of sufficient intracellular iron by the ferric uptake regulator (Fur) [196]. The ECM also helps maintain this balance in biofilms by storing iron sequestered within the three EPS [195]. PVD has a dual role, also acting as a signalling molecule for the production of ETA, an endoproteinase (PrpL) and PVD itself. When the ferrisiderophore complex interacts with FpvA, it is initiated a signalling cascade by interacting with the anti-σ factor FpvR, allowing the expression of two regulators (PvdS and FpvI). Moreover, an intrinsic relationship exists between the Pqs QS system and iron levels, since the major regulator PvdS controls the expression of PqsR, and thus PQS synthesis [21]. For its part, pyochelin can cause oxidative damage and inflammation, especially in the presence of pyocyanin [193].

### 2.9. Quorum Sensing

Quorum sensing is critical for regulating several genes, allowing for cell–cell communication and adaptation to environmental changes [197]. *P. aeruginosa* has four QS systems (Las, Rhl, Pqs, and Iqs) that are interconnected hierarchically: the Las system is at the top of the signalling hierarchy, positively controlling the expression of the other three systems. Similarly, the Iqs system has a stimulatory effect on Pqs, and this on Rhl system, whereas Rhl regulates Pqs negatively [171,197,198,199]. This QS network is highly adaptable and capable of responding to external stressors, providing *P. aeruginosa* with an extraordinary flexibility [197].

#### 2.9.1. Acyl-Homoserine Lactone QS Systems: Las and Rhl

LasR and Rhl represent the most dominant regulatory circuits. In the Las system, LasI is the autoinducer (AI) synthase that mediates N-3-oxododecanoyl-L-homoserine lactone (C12HSL) synthesis. LasI binds to the transcriptional activator (LasR), creating a complex with multimeric forms that specifically regulates the transcription of virulence genes involved in acute infections and host cell damage (LasA, LasB, AprA, PVD, and ETA) (Figure 1b). It also induces the production of the AI, creating a positive autoregulatory loop [21,200]. The RsaL inhibitor is responsible for LasI and C12HSL synthesis repression [197]. The Las system also suppresses the production of Pel exopolysaccharide [201], affects T6SS formation along with Rhl and Pqs systems [202,203], and induces apoptosis in airway epithelial cells and degrades their tight junctions [204,205]. C12HSL also helps bacterial survival by producing host immune cell death [206]. Importantly, PYC and C12HSL increase the number of persister cells in *P. aeruginosa* populations, which may be responsible for the recalcitrance of chronic infections [207]. Similarly, RhlI synthesises the AI of this system, N-butyryl-L-homoserine lactone (C4HSL), which forms a complex with the activator protein (RhlR) [200]. This circuit mainly enhances the production of RL, but also of AprA, LasB, cyanide, PVD, and PYC [200]. It also regulates the production of LecA lectin, influencing biofilm formation [208], and represses genes implicated in T3SS assembly and function [104,209].

#### 2.9.2. The Quinolone QS System: Pqs

*P. aeruginosa* produces numerous alkyl-4(1H)-quinolones (AQ), including 2-heptyl-hydroxy-1H-quinolin-4-one (PQS) and its precursor 2-heptyl-4(1H)-quinolone (HHQ), the most commonly AQ associated with QS. They are synthesised by enzymes encoded in *pqsABCDE*, *phnAB*, and *pqsH* gene clusters, and both PQS and HHQ are recognised by the cognate regulator protein (PqsR or MvfR) [210] (Figure 1b). Like the Las system, the Pqs system creates a positive feedback loop binding to the promoter of *pqsABCDE*, leading to the production of PqsE, the major virulence effector of the quinolone system [210]. This protein, together with the Rhl system, is involved in pyocyanin synthesis regulation. Additionally, it positively regulates the expression of genes related to iron starvation, efflux pumps involved in antibiotic resistance, and the biosynthesis of hydrogen cyanide, RL, elastase, and extracellular chitinase [211]. Furthermore, the Pqs system mediates eDNA release, essential for the creation of stable and mature biofilms [208]. Apart from being a QS signalling molecule, PQS also acts as a mediator in iron acquisition, cytotoxicity, and OMV biogenesis; suppresses IL-2 and IL-12 secretion; and stimulates neutrophil chemotaxis, ROS, and TNF-α generation [210].

#### 2.9.3. The Novel QS System: Iqs

This integrated QS system was discovered more recently and uses a new type of signal molecule: 2-(2-hydroxyphenyl)-thiazole-4-carbaldehyde (IQS). To date, its cognate receptor is unknown [212]. In addition to monitoring bacterial density, Iqs also detects phosphate limitation, a common stress during infection, to regulate virulence factor production [199]. Moreover, it may partially control the functions of the Las system and, when disrupted, the production of pyocyanin, rhamnolipids, and elastases decreases [199]. Finally, IQS inhibits host cell growth and stimulates apoptosis in a dosage-dependent manner, subverting the host DNA damage repair [213].

## 3. CF Lung Environment

*P. aeruginosa* elicits a robust acute host inflammatory response; however, it manages to persist within the airways [1]. Its tremendous metabolic flexibility allows it to readily adapt to airway conditions, which has been widely studied in people with CF where *P. aeruginosa* chronic infection is the leading cause of lung function decline [214]. CF is the most common autosomal recessive genetic disorder among Caucasians. It is caused by mutations in the CF transmembrane conductance regulator (CFTR), which is responsible for chloride ion transport across apical membranes of epithelial tissues [215,216]. Hence, CFTR deficiency leads to diminished chloride transport and increased sodium transport through ENaC, resulting in a dehydrated airway surface liquid (ASL) and the production of mucopurulent secretions that are difficult to clear [215]. The CF lung environment has been widely described by other authors [217,218,219,220], so this review will focus on the main aspects specifically associated with *P. aeruginosa* respiratory infections.

**Figure 2:**
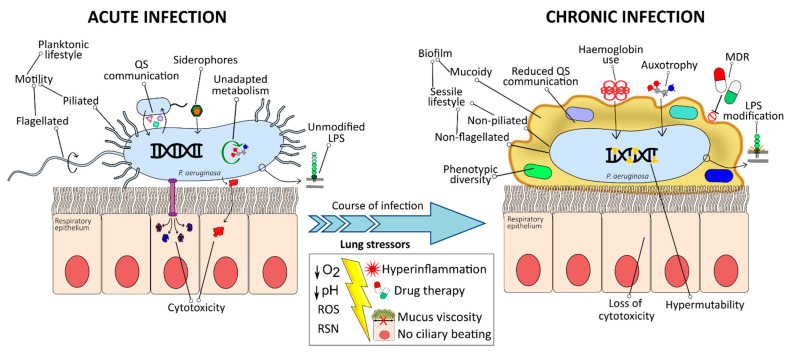
Representation of *P. aeruginosa* adaptation to the cystic fibrosis (CF) lung over the course of infection. In early stages, isolates are fully equipped with virulence factors that allow respiratory tract colonisation and lung injury. The stressful environment of the CF airway drives *P. aeruginosa* pathoadaptative changes that enable long-term colonisation and establishment of recalcitrant infections.

The dehydration of ASL leads to many changes in the airways, including effectively poor mucocilliary clearance, low pH, and impaired antimicrobial and immune response mechanisms (Figure 2). Cilia cannot effectively move mucus out of the lung, facilitating chronic bacterial infection by *P. aeruginosa* [221]. The acidic environment of the CF airways results in the improper folding of the carbohydrate side chains of mucins, hampering their ability to bind foreign particles and making them more likely to bind to the cell-tethered mucins MUC1 and MUC4, gluing the mucous layer to the epithelium and preventing mucocilliary clearance [142]. The low pH is also associated with an altered O-glycosylation and sulfation of the airway mucins, mainly due to the alkalization of the cell compartments in CF. This unique phenotype of sputum O-glycosylation increases the ability of bacterial pathogens to adhere to and colonise the host’s respiratory tract [222]. *P. aeruginosa* attaches preferentially to asialoglycoprotein; as a consequence, the malfunctioning of CFTR could increase *P. aeruginosa* CF airways colonisation [223].

The inhibition of antimicrobial peptides also occurs due to acidic conditions. Pezzulo et al. demonstrated that although the antimicrobial composition of the ASL was similar in CF and non-CF pigs, the CF pigs’ airways showed reduced efficiency in bacterial killing, suggesting that the observed acidic environment may contribute the lack of activity of antimicrobial molecules [224,225]. Another effect of the acidic environment is the delay in neutrophil apoptosis and the suppression of IFN-γ production by T helper (Th)1 cells [226]. Recruitment of neutrophils can lead to a reduction of O_2_ in the airway mucus due to the intensive consumption of O_2_ by polymorphonuclear leukocytes (PMN) for superoxide and nitric oxide production. PMNs exert a bacteriostatic effect on aggregate bacteria since the growth rate of *P. aeruginosa* in CF mucus is inversely correlated with the amount of PMNs. Given that the most effective production of adenosine triphosphate (ATP) by *P. aeruginosa* occurs by aerobic respiration, the lack of O_2_ may contribute to the inactive and therefore tolerant state of this pathogen in mucus [227].

The C-terminal tail of CFTR appears to bind to the N-terminal of tumour suppressor phosphatase and tensin homolog (PTEN) and may promote the hyperinflammatory state in the CF airways. Such interaction may be necessary for PTEN membrane localization. In a CF mouse model, Riquelme et al. reported that the dysfunctional PTEN associated with CF may suppress the activity of mitochondrial succinate dehydrogenase, leading to increased secretion of succinate in the airways. *P. aeruginosa* easily metabolised this succinate, promoting the colonisation of the respiratory tract by this pathogen, but not by *S. aureus*. A continuous adaptation to succinate was observed in longitudinal isolates from a CF patient. Succinate-adapted *P. aeruginosa* suppressed the immune response in human monocytes and mice, which may promote persistence in CF patients [17,228].

The frequent use of antibiotics in CF can represent one of the biggest challenges bacteria face in their struggle to survive. Antibiotic use is the primary driver of decreasing bacterial diversity in the respiratory tract of CF patients. Airway bacterial diversity peaks in young adulthood and then declines with advancing age and disease progression [229]. The decline in lung function has been associated with reduced microbial diversity, and hence a predominance of opportunistic pathogens. Quin et al. observed that CF metabolism existed in two states: one in severely diseased patients who had higher molecular diversity and more *P. aeruginosa* and another in patients with better lung function, lower metabolite diversity, and fewer pathogenic bacteria. They concluded that in cases of severe CF, there is an amino acid-rich environment due to proteolysis by host enzymes which become dominated by *P. aeruginosa* as amino acid richness provides the pathogen its preferred carbon source [230].

The study of the respiratory tract environment in CF is complex. In addition to the impact of CFTR, “modifier genes” affect the CF phenotype and generate variability in pulmonary severity among patients [231]. Some of these genes also impact *P. aeruginosa* infections in CF patients. Among them is *SLC6A14*, which is expressed in respiratory epithelial cells and transports L-arginine out of ASL. Di Paola et al. suggested that *SLC6A14* plays a role in modifying the early stages of *P. aeruginosa* infection in the airways by altering the level of L-arginine in ASL, which in turn affects *P. aeruginosa* adhesion [232]. Other modifier genes associated with *P. aeruginosa* infection include C3 and HMOX1. The HMOX1 level is elevated in CF patients and is responsible for cytoprotective effects against *P. aeruginosa* infection; thus, polymorphisms in the HMOX1 gene could result in a deficient inhibition of tissue damage due to *P. aeruginosa* infection, leading to increased disease severity in CF patients. On the other hand, C3 is involved in the complement system, which is relevant in the innate immune response against *P. aeruginosa* [233].

The added complexity in CF airways is the CF gender gap, well described in other reviews [234,235,236]. Women with CF are at increased risk for the mucoid conversion of *P. aeruginosa*, which contributes to a sexual dichotomy in disease severity. Chotirmall et al. concluded that estradiol and estriol induced alginate production in PAO1 and clinical isolates obtained from patients with and without CF. After prolonged exposure to estradiol, *P. aeruginosa* adopted an early mucoid morphology. Interestingly, a review of the CF Registry of Ireland suggested that the use of oral contraceptives was associated with a decreased need for antibiotics [237]. Moreover, Tyrrell et al. demonstrated for the first time that estrogen exacerbates *P. aeruginosa* virulence and enhances bacterial interactions with CF bronchial epithelium. Estrogen also increased biofilm formation of PAO1 which became more adherent to normal and CF bronchial epithelial cells [238].

*P. aeruginosa* has managed to tailor its extensive arsenal of virulence factors to adapt to this hostile CF lung. The adaptations that *P. aeruginosa* undergoes to survive in this type of hostile environment are described below.

## 4. Bacterial Adaptation within the Lung

Despite the host immune response and the antimicrobial therapy, *P. aeruginosa* can persist for decades in the respiratory tract of patients, where the bacteria undergo several convergent evolutionary changes, resulting in recurrent genomic and phenotypic adaptations that promote bacterial survival by attenuating virulence and avoiding immune recognition. Consequently, host-adapted variants and initial strains are considerably different [239,240] (Figure 2). For example, a loss of virulence in the *Galleria mellonella* model (and pyocyanin) was observed in three independent series of sequential *P. aeruginosa* strains isolated over time of chronic infection [241].

### 4.1. Emergence of Hypermutators

Hypermutable microbes display increased spontaneous mutation rates (up to 10,000-fold) due to defects in DNA mismatch repair (MMR) or error avoidance (GO) systems [242]. Antibiotics and host environment both select for mutator strains, which are rarely found during acute infections [242]. Hypermutability is necessary for effective lung adaptation and long-term persistence, as it allows strains to increasingly accumulate adaptive gene mutations that involve inactivation of certain virulence factors and increased drug resistance [243]. In fact, deficiency in the MMR system has been linked to the emergence of CF-related phenotypes [244]. In the CF airways, the prevalence of *P. aeruginosa* mutators is extremely high, approximately 10–30% of isolates [242], increasing during the course of the chronic infection [245]. *P. aeruginosa* isolates from CF and COPD patients show a defective MMR system, mainly caused by mutations in *mutS* or *mutL*, and less frequently in *uvrD* (*mutU*), whereas few mutations have been detected in the GO system genes (*mutM*, *mutY* and *mutT*) [242].

### 4.2. Phenotypic Diversity and Morphology Variants

*P. aeruginosa* shows an exceptional degree of phenotypic diversity in the lung environment, inevitably leading to the co-existence of subpopulations [244,246,247,248]. Such diversity may be driven by hypermutability; geographical isolation and spatial heterogeneity in the lung may be key factors in the diversification process [248,249]. Small colony variants (SCV) are frequently isolated from chronically infected respiratory tracts of CF, COPD, or mechanically ventilated patients. They appear due to prolonged antibiotic therapy and show an auto-aggregative and slow-growing behaviour, hyper-piliation, overproduction of one or more EPS, enhanced biofilm formation ability, and antibiotic resistance, being associated with poor clinical outcomes [250]. Another phenotype that causes severe infections is the rugose small colony variant (RSCV), characterized by excessive amounts of Pel and Psl and hyper-biofilm forming ability due to flagellar mutations and others leading to c-di-GMP overproduction [251,252]. This phenotype provides *P. aeruginosa* with augmented tolerance to host defences and elicits a robust but ineffective inflammatory response from neutrophils, which likely contributes to host tissue damage [252]. Persister cells, which can restore a *P. aeruginosa* population after antibiotic treatment, were found in 56% of CF patients, which emerged through different genetic routes [253].

### 4.3. Mucoid Phenotype Switch and Sessile-Biofilm Lifestyle

Mucoidy is a hallmark of the transition from acute to chronic lung infections. Later isolates show a mucoid phenotype due to the overproduction of alginate [240], which are associated with poor prognosis, lung function decline, severe bronchiectasis, and increased mortality in respiratory patients [254]. The stressors in the inflammatory lung of CF and COPD patients drive the emergence of inactivating mutations in the *mucA* gene, which encodes an anti-σ-factor that sequesters AlgT, repressing the expression of *algD* operon [20,254,255,256]. Alginate-overproducer strains overcome clearance by antibiotics and the immune response, and offer a survival advantage to bacteria by providing physical protection, trapping essential nutrients and downregulating virulence factors (flagella, pili, LPS, T3SS, and QS systems), promoting the biofilm lifestyle [257]. Although mucoid strains produce less Psl than non-mucoid strains, this EPS still mediates adhesion to human airway cells, protects from opsonophagocytic killing by complement components and contributes to the establishment of biofilms [258]. Whether T6SS is up- or downregulated in mucoid strains remains unclear. One study detected reduced levels of T6SS effectors in a non-mucoid strain [259], while another study showed increased expression in a mucoid isolate [241]. Moreover, T6SS has recently been associated with biofilm-forming strains [260].

Late non-mucoid isolates have also been recovered from the CF lung. These isolates are revertants of mucoid isolates rather than wild-type isolates since they show mutations in the *mucA* gene, and such reversion may arise from non-silent mutations in *algT*. This likely occurs because the production of alginate is a high-energy requiring process for the bacterium [185]. Mixed populations of mucoid and non-mucoid variants also exist in CF lungs, being an advantage for evasion of host innate antimicrobials [261].

### 4.4. Loss of O-Antigen and Structural Modifications of Lipid A

During chronic infections, LPS undergoes various structural changes as a consequence of the emergence of non-synonymous mutations in genes encoding enzymes involved in its biosynthesis [20,27,240,262]. Isolates from chronic infections frequently show loss or reduced production of O-antigen (rough colony phenotype), diminishing bacterial immunogenicity, and transformation into non-typable strains [27,29]. Moreover, mucoid strains express reduced levels of Wzz2 (the very long O-antigen chain length control protein), resulting in shorter OPS chains [263]. Usually, OSA is lost, contributing to antimicrobial resistance (AMR), while CPA expression is more stable in the CF lung and becomes the major LPS antigen overtime [28,29], likely due to its importance in biofilms and recalcitrant infections.

Three common modifications in lipid A are also encountered in isolates recovered from CF airways: (i) addition of an O-linked secondary palmitate to the OH group of sugar, observed in the majority of CF isolates; (ii) the addition of aminoarabinose to either or both of the terminal phosphates, encountered in less than half of CF isolates; and (iii) the modification of the lipid A acylation patterns [27,34]. Acylation patterns can differ, with some isolates underacylated, whereas others present additional acyl chains; for instance, hepta-acylation was related to severe lung disease [264,265]. This remodelling protects *P. aeruginosa* against host innate defences by further reducing the permeability of the OM to host AMPs, dampening host inflammatory responses, and modulating TLR4-MD2 receptor recognition [27].

### 4.5. Lack of Motility and Non-Flagellated, Non-Piliated Phenotype

Since the flagellum is a potent activator of inflammatory responses and subject to detection by host receptors (TLR5 pathway and NLRC4 inflammasome), *P. aeruginosa* tends to lose it over the course of infection through different mechanisms [240,262]. It is genetically regulated by AlgT-dependent and QS-independent mechanisms [266,267]. Genetic mutations in regulatory genes have also been detected in late isolates [20,268,269]. Moreover, flagellar mutants are highly selected for by the CF environment, which overproduce EPS and contribute to biofilm formation [251]. It also depends on proteolytic control: secreted LasB and AprA degrade flagellin [148], and neutrophil elastase cleaves the flagellar hook, leading to intracellular accumulation of FlgM protein and repression of flagellin synthesis [270]. Loss of flagella directly entails loss of motility, which avoids inflammasome activation [269], inhibits superoxide production and NET formation [271], and confers a marked resistance to phagocytosis, independent of flagellar expression [272,273]. In addition to flagella-driven motilities (swarming and swimming), twitching motility is also lost due to the downregulation of T4P. Mutations in *pilB* or deletion of *pilQ* genes may contribute to non-piliation. Nonetheless, most of CF isolates exhibit *rpoN* mutations, provoking the loss of both pili and flagella [20,274,275]. Recently, AlgT and AmrZ were shown to be involved in *pilA* gene repression, thereby inhibiting pili formation and twitching motility [276].

### 4.6. Selection against T3SS and Loss of Cytotoxicity

The injection of destructive cytotoxic effectors is not compatible with bacterial persistence [117], and the loss of T3SS hinders inflammasome activation [269]. Therefore, while T3SS plays a dominant role in acute infections, its absence is advantageous for *P. aeruginosa* in chronic infections [240]. Isolates from chronically infected patients presented accumulated mutations with functional effects in PopB, PscI, and ExsA encoding genes [20,269]. Additionally, high levels of c-di-GMP and the MucA/AlgU signal transduction system repress the expression of T3SS proteins [109,277,278].

### 4.7. Reduced Communication Systems

Loss of QS is frequently encountered in isolates from later stages in the CF infection [240,262]. Mucoid *mucA* mutants downregulate the three major QS systems [279]. Chronically infected patients harbour *lasR* mutants that show a reduced production of C12HSL [20,279,280,281,282,283]. As the Las system is at the top of the regulatory hierarchy, deletion in this system may also reduce the production of quinolones by the Pqs system [279,283]. Loss-of-function mutations are also found in genes encoding components of the Rhl system (*rhlR* and *rhlI*) [20] and in other genes like *gacS* and *retS*, which are part of the two-component GAC regulatory system that controls transition from acute to chronic infection [5]. Considering that the expression of many virulence factors that participate during the acute stage is orchestrated by QS, most of them are lost during the course of the infection [239]. Lytic enzyme-deficient strains are repeatedly isolated from chronically colonised CF and COPD patients [283]. Because bacterial protease-dependent cytokine degradation is lost in *las* mutants, exaggerated host inflammatory responses in respiratory epithelial cells are generated, characterised by accumulation of proinflammatory cytokines and neutrophil recruitment [284], likely contributing to *lasR* mutants’ association with poor lung function [282]. Other QS-dependent products, such as PYC or ETA, are also decreased [262,279].

### 4.8. Specialised Metabolism

CF mucus is abundant in amino acids and other nitrogen sources. Because amino acid production is extremely costly and they are available in the host, *P. aeruginosa* loses the ability to synthesise amino acids via non-silent mutations in their biosynthetic pathways [285]. Among CF isolates, methionine, leucine, and arginine auxotrophies are the most commonly encountered. This adaptation may be a double-edged sword because, while it contributes to high fitness in the CF airways, it also limits the potential to move to other environments, where nutrients are scarce [285]. A recent study showed that a CF isolate was only able to use purines and DNA as carbon sources, likely as an adaptation to the availability of eDNA in the CF environment [286].

### 4.9. Change of Iron Uptake Strategy

Early in the airway colonisation, *P. aeruginosa* produces pyoverdine for acquisition of iron; however, PVD-deficient strains increase with longer times of colonisation [193]. Iron uptake is vital and necessary for correct biofilm development [195], thus, *P. aeruginosa* adapts towards haemoglobin utilisation within the host, instead of using siderophores. This is caused by mutations in the Phu system encoding genes, such as *phuR*, *phuT,* and *phuUV* [287]. The deletion of surface TonB-dependent receptors of the siderophores has also been detected [288].

### 4.10. Acquisition of Antibiotic Resistance

Antibiotic resistance is another hallmark of chronically colonising isolates from the CF lung [20,289]. *P. aeruginosa* is intrinsically resistant to antibiotics due to the particularly low permeability of its OM and the presence of drug efflux pumps, porins, and β-lactamases [26,142]. However, pathoadaptive mutations and micro-indels in their encoding genes arise as a result of intense drug therapy [20,290,291], leading to overexpression of efflux pumps, altered antibiotic targets, hyperproduction of β-lactamases (i.e., AmpC), and further reduced OM permeability due to porin loss [26,245,256]. This mutation-mediated antibiotic resistance strongly correlates with hypermutators [245]. Moreover, adaptations such as overproduction of alginate and biofilm formation [78], LPS modifications (lipid A aminoarabinosylation and loss of OPS) [28], and QS mutations [281] all contribute to antibiotic resistance. Consequently, *P. aeruginosa* chronic infections usually develop a multidrug resistance phenotype, thereby evading bacterial eradication.

## 5. Genomic and Phenotypic Approaches to the Study of *P. aeruginosa* Adaptation within the CF Lung

The ubiquity of *P. aeruginosa* may be closely related to the high genome plasticity. *P. aeruginosa* has a genome of approximately 5.2 to 7 Mbp [292,293,294], with 4000 genes within the “core genome”. The complete set of genes among different *P. aeruginosa* strains varies from 10,000 and 40,000 genes, and, interestingly, their arrangement in the genome may differ between strains; therefore, identification of suitable regions for genetic markers is difficult [292,294,295]. There is a lot of detailed information about the *P. aeruginosa* genome, transcriptome, and proteome available from several databases: (i) the *Pseudomonas* Genome Database [296]; (ii) PseudoCyc [297]; (iii) SYSTOMONAS [298], KEGG [299], PubChem [300], and HMDB [294,301].

Developments in genomic sequencing enabled a greater understanding of the adaptation and evolution of *P. aeruginosa* in chronic CF lung infections, revealing high levels of co-existing genetic and phenotypic diversity, including clinically important traits (Table 2) [5]. Whole-genome sequencing of *P. aeruginosa* isolates obtained longitudinally from CF patients provided evidence that during long-term infection, *P. aeruginosa* undergoes adaptive processes leading to the accumulation of mutations in the infecting strains [216].

In most CF patients, the primary colonising bacterium (clone) persists and dominates for long periods. Rarely, an incoming strain of *P. aeruginosa* may compete sufficiently to displace the indigenous population. Interestingly, strain genotyping has shown that when such cases occur, the invading strain came from another CF patient chronically infected with that strain [216,287,302,308,309]. It was thought that transmission of strains between CF patients only occurred with very close contact between patients [295,310,311,312]. However, this hypothesis was first refuted with the report of a MDR *P. aeruginosa* clone in a Danish paediatric cohort [295,313,314]. The identification of the Liverpool epidemic strain (LES) strain exploited molecular techniques to demonstrate for the first time that patients shared the same transmissible strain [315]. In general, the majority of first *P. aeruginosa* infections in early childhood occur with unique, nonclonal strains [295,316,317], while shared strains are observed among older patients [295,317,318,319,320,321].

Despite the transmissibility of certain *P. aeruginosa* strains among CF patients, significant diversity within genetically related colonising *P. aeruginosa* clones has been demonstrated within individuals. Diversity at the genomic level is represented by point mutations, insertions, and even large-scale deletions, leading to the emergence of clades, which persists depending on how much they manage to compete and adapt to survive in the complex airway environment of CF patients (Section 3, Figure 2) [295,309]. Thus, sequential isolates of the same ancestral strain from a CF patient can demonstrate great phenotypic heterogeneity [239,322,323], which makes the direct comparison of phenotypes between specific strains difficult [247,295,322,324] and must be taken into account when comparing traits of individual *P. aeruginosa* clones [295]. Clonal strains showing a high degree of variance across multiple phenotypes coexisted in a singly colony morphotype from a patient [247].

Among the most mutable genes identified in longitudinal isolates from CF patients are those linked to a biofilm-associated lifestyle (*mucA, algU,* and *morA*), decreases in antibiotic susceptibility (*mexZ, nfxB, mexR, gyrA, gyrB,* and *mpl*), reduced virulence factor production (*ykoM* and *mpl*), and different regulatory systems (*rpoN, nfxB, mexR, gacA,* and *gacS*), including QS, in different patient lineages despite different clonal backgrounds [216]. The sequencing of bacterial genomes has been a key to demonstrate the evolution of bacterial clones through mutational changes in pre-existing genes, a mechanism also known as pathoadaptive mutation [302,325]. This is especially evident with the sequencing of *P. aeruginosa* genomes from CF patients. For example, the sequencing of 474 longitudinal clinical isolates of *P. aeruginosa* from 34 children and young adults with CF identified 36 *P. aeruginosa* lineages and convergent molecular evolution in 52 genes. A succession of mutations in key regulatory networks were also identified, indicating these are important for *P. aeruginosa* adaptation. This highlights the importance of clinical collections from chronically infected patients in understanding the convergence and evolutionary contingency of pathogens in vivo for the design of future therapeutic strategies [302]. For example, a well characterised panel of strains which includes three series of sequential isolates from CF patients consistently showed reduced virulence, O antigen expression, and pyocyanin production in later infection isolates [256,262].

Genomic studies have also provided insights into mechanisms of antibiotic resistance. Greipel et al. examined 17 antimicrobial susceptibility and resistance loci in an international strain collection of 361 *P. aeruginosa* isolates from 258 CF patients, identifying 1112 sequence variants that were not present in the genomes of strains representative of the 20 most common clones in the global *P. aeruginosa* population. A high frequency of variants was observed in *spuE, mexA, gyrA, rpoB, fusA1, mexZ, mexY, oprD, ampD, parR, parS,* and *envZ* (*amgS*), which appear to be involved in the response of *P. aeruginosa* populations to antimicrobial load in CF. Interestingly, the highest relative proportions of SNPs that were absent from the pangenome reference were found in *fusA1A2, mexA*, and *pagL* which code for proteins involved in translation, transport, and modification of LPS, respectively. Thus, suggesting that de novo mutations may play an essential role in the adaptation of *P. aeruginosa* populations in individual CF lungs in an attempt to escape antimicrobial pressure [303].

Another study showed the mutational profile of the resistome of a hypermutator lineage of *P. aeruginosa* by performing longitudinal and cross-sectional analyses of isolates collected from a CF patient over 20 years of chronic infection, demonstrating an accumulation of thousands of mutations. Mutations in antibiotic resistance genes were positively selected, driven by antibiotic treatment. The infection progressed towards the establishment of a population consisting of genotypically diversified co-existing sublineages, all of which converged towards multi-drug resistance. Importantly, these sublineages arose by parallel evolution through distinct evolutionary pathways, affecting genes in the same functional categories. The *ampC* and *ftsI* genes, encoding β-lactamase and penicillin-binding protein 3, respectively, were among the most frequently mutated genes [243].

In vitro studies utilized genomics to investigate *P. aeruginosa* evolution and acquisition of AMR. For example, Ahmed et al. demonstrated that the pathways of developing Ciprofoxacin (CIP) resistance are growth mode-dependent, and they suggested evolved phenotypic and genotypic changes that paralleled the evolution of CIP resistance. Cross-resistance to β-lactam antibiotics was associated with mutations in genes involved in cell wall recycling (*ftsZ, murG*) and could also be explained by mutations in TCA cycle genes (*sdhA*) and genes involved in arginine catabolism. Interestingly, the set of identified mutated genes overlaps with a large number of pathoadaptive genes previously reported in *P. aeruginosa* isolates from CF patients [304]. Furthermore, pyomelanin-resistant mutants frequently coexist with other morphotypes in CF patients [326]. Chromosomal deletions included *hmgA* [327] and *galU,* [328]; *mexXY*, contributes to intrinsic aminoglycoside resistance in *P. aeruginosa* [329].

Genomic differences observed in different clones within the same CF patient are not always reflected in phenotypic studies. La Rosa et al. analysed 26 clinical isolates of *P. aeruginosa* belonging to three different clone types, exhibiting naïve, intermediate, and adapted phenotypes, sampled from a single CF patient over an 8-year period of infection. Evolution within the patient involved convergent metabolic specialisation characterised by loss of non-essential metabolic functions, independent of clone type, genomic composition, or mutation pattern. Thus, different combinations of genetic and regulatory changes converge on common metabolic adaptive trajectories leading to metabolic specialisation within the host [305]. In addition, Oakley et al. analysed the experimental evolution of *P. aeruginosa* in response to OligoG CF-5/20, an inhaled alginate oligomer therapy currently in phase IIb/III clinical trials in CF patients. They used a biofilm model for 45 days (∼245 generations). Mutants isolated after OligoG CF-5/20 treatment typically exhibited reduced biofilm formation capacity and an altered motility profile. However, genotypically, OligoG CF-5/20 did not provide any selective pressure on genomic mutations within morphotypes [330]. Experimental evolution of *P. aeruginosa* biofilms over 600 generations showed a higher mutation rate in biofilms over planktonic populations and diverse colony morphologies within an individual biofilm [331].

Analysis of genome-wide extended multilocus sequence typing (wgMLST) of four *P. aeruginosa* strains of environmental and clinical origin, compared to the wgMLST of PAO1 and PA14 type strains, showed no genomic feature common between the strains. However, ten loci were highly discriminatory in the context of *P. aeruginosa* virulence and evolution. Two of the *loci* identified (*exsA* and *rsmN*) were master regulators involved in the expression of the T3SS and expression of QS-regulated virulence traits. A third *locus* was a type III effector protein (HopJ). Thus, they showed that the establishment of pathogenic interactions, and in particular the activity of the T3SS, is a key feature of *P. aeruginosa* [306].

Bartell et al. highlighted the value of classical phenotype-based investigations to complement genomic approaches. Using statistical modelling, they examined eight infection-relevant phenotypes of 443 longitudinal *P. aeruginosa* isolates from 39 young CF patients over 10 years. They identified emergent patterns of bacterial phenotypic change across the patient cohort that deviate from expected evolutionary trajectories, estimating a period of initial rapid adaptation during which bacteria move from a “naïve” to an “evolved” phenotypic state. They proposed new associations between observed phenotypic phenomena and genetic adaptation. Multi-trait modelling can map complex, patient-specific evolutionary trajectories that will allow understanding pathogen persistence and how to prevent it [307].

Other interesting approaches to adaptation are those that have investigated the interactions between *P. aeruginosa* and other pathogens found in the lungs of CF patients. For example, sequencing of clinical *S. aureus* isolates from the lungs of CF patients showed differences in their interactions with *P. aeruginosa* ranging from being very sensitive to *P. aeruginosa* to being completely tolerant to it. They identified three distinct phenotypic groups of *S. aureus* based on their survival in the presence of nonmucoid PAO1 and its mucoid derivative. Finally, adaptation has also been evaluated with murine studies of chronic infection. Vanderwoude et al. found that genes previously implicated in *P. aeruginosa* pathogenesis (*lasR, pilR, fleQ, rpoN,* and *pvcA*) contained mutations during the course of evolution in a chronic infection wound model, with selection occurring in parallel in all lines of evolution [332].

Genomics has been key to the study of the evolution of *P. aeruginosa* within the CF environment and the transmissibility of strains between patients. It allows a better picture of how *P. aeruginosa* genes regulating virulence factors and AMR are conserved or acquired. The complexity and plasticity of *P. aeruginosa* genome give it a great diversity that hinders the understanding of *P. aeruginosa* persistence, hampering the development of therapies against this challenging pathogen.

## 6. Conclusions

The extensive repertoire of virulence factors combined with its adaptability facilitates *P. aeruginosa* in being the most prevalent pathogen in the CF airways, persisting within the host and causing chronic and recalcitrant infections despite the hostile environment of the CF airways. The number of virulence factors and variety of AMR mechanisms expressed by *P. aeruginosa* together with its complex regulatory networks are impressive. They help *P. aeruginosa* to evade the host immune system, as seen with LPS and OMPs, and/or enable the secretion of exotoxins and proteolytic proteases. Its robust biofilm-forming capacity protects it from antibiotics or other agents enabling *P. aeruginosa* to persist in inhospitable environments, while its flagellar system allows it to colonise different niches. Furthermore, its secretion systems enable it to inject toxins into both prokaryotic and eukaryotic cells, allowing *P. aeruginosa* not only to survive the immune system attack but also to compete with other microorganisms. Importantly, these traits collectively give *P. aeruginosa* tremendous plasticity, utilising different regulatory pathways for the same phenotype and turning it into an extremely adaptive pathogen. Therefore, it is not surprising that *P. aeruginosa* survives in the hostile CF lung environment overcoming immune response mechanisms.

In recent years, the sequencing of sequential and longitudinal isolates from CF patients has provided valuable information on how *P. aeruginosa* manages to evolve and persist in the host by favouring some virulence factors over others. It also enabled the identification of persistent or transmissible clones, highlighting some adaptation traits such as the emergence of hypermutators, overproduction of alginate, loss of flagellum and pili, loss of cytotoxicity, reduction in communication systems (QS), and acquisition of antibiotic resistance, among others. Genomics facilitated the elucidation of the adaptive mechanisms of *P. aeruginosa*, but its integration with phenotyping studies will support the full interpretation of the evolutionary dynamics of the pathogen within the host. Overall, it is evident that to tackle a pathogen as challenging as *P. aeruginosa,* it is necessary to be well informed of the weaponry it possesses, which is why comprehensive knowledge of its virulence factors and its behaviour within the lung is a priority for the design of any therapy against *P. aeruginosa* infections.

## Figures and Tables

**Table 1:** Summary of the main *P. aeruginosa* outer membrane proteins (OMPs), their homologue in *Escherichia coli* when known, and the function they play in *P. aeruginosa* virulence.

OMP in *P. aeruginosa*	Homolog in *E. coli*	Function	Ref.
OprF	OmpA	Cell integrity maintenance	[42,43,44]
		Ion and saccharide acquisition	[26]
		Peptidoglycan binding	[42,44]
		Diffusion channel (toluene, siderophores, nitrates, nitrites)	[42]
		Adhesion (alveolar epithelial cells and other bacteria)	[42,43]
		Regulation of other virulence factors	[42,45,46,47,50]
		Immune system sensor	[43,47,51,52]
OprH	OmpW family	Protein binding (SP-A and laminin)	[53,54]
		Aminoglycoside and polymyxin resistance	[42]
		Transport (hydrophobic molecules, amino acids, iron, and cations)	[42,50]
OprD (OccD1)	OmpF	Laminin binding	[42,54]
		Carbapenem resistance	[42]
		Molecule transport (amino acids, peptides, gluconate)	[42]
OprG	OmpW family	Laminin binding	[42,54]
OprQ (OccD6)		Fibronectin binding	[55]
		Adhesion (epithelial cells)	[55]
OprL	Pal	Cell integrity maintenance	[24]
		Protection against oxidative stress	[56]
OprI	Lpp	Cell integrity maintenance	[24]
BamBDE		OM biogenesis	[24]
LptE		OM biogenesis	[24]
OprJMN		Antibiotic resistance	[24,57]
OmpBEG		Antibiotic resistance	[24,57]

**Table 2:** Examples of *P. aeruginosa* genomic evolution and adaptation studies.

Type of Study	Source of Isolates	Main Findings	Frequently Mutated Genes	Function of Identified Mutated Genes	Ref.
In vivo evolution study using whole genome sequencing	474 longitudinal CF clinical isolates from 34 children and young individuals.	36 lineages with convergent evolution in 52 genes	*asR*, *mexA*, *mexS*, *nex, yecS, algU, gyrA, gyrB, mexB, oprD, pela,* and *rbdA*	Host adaptation, AMR, and loss of extracellular virulence factors	[302]
In vivo evolution study of 17 AMR loci	361, independent CF isolates collected from 30 CF centres.	1112 sequence variants not present in the 20 most common PA clones	*spuE, mexA, gyrA, rpoB, fusA1, mexZ, mexY, oprD, ampD, parR, parS, and envZ (amgS),* and *pagL*	Unrelated. Translation, transport, LPS modification, and AMR	[303]
In vivo longitudinal and evolution analysis	14 isolates from the same clonal lineage of a CF patient (20 years of the infection).	Evolution towards purifying selection. Different evolutionary pathways affecting genes of the same functional categories	*ampC, ftsI*	Codification of β-lactamase and penicillin-binding protein 3 (AMR)	[243]
In vitro biofilm and stationary-phase planktonic culture evolution study	57 CIP-evolved populations and 35 control.	CIP-resistance development depends on bacterial lifestyle	*ftsZ*, *murG, sdhA*	Cell-wall recycling, TCA cycle, and arginine catabolism	[304]
Real-time in vivo evolution, metabolic and genomic study.	26 from a single CF patient (8 years of infection).	Convergence at the phenotypic level but different mutational patterns	Not specified (functional grouping)	Amino acid transport and metabolism, defense, signal transduction and translation	[305]
In vivo genome analysis (wgMLST)	2 environmental, 1 veterinary and a CF clinical isolates with a defective Las QS system	Identification of ten highly discriminatory *loci* between the studied strains and the PAO1 and PA14 strains	*exsA*, *rsmN*, and *hopJ*	T3SS and QS-regulated virulence traits.	[306]
Screening of 8 infection-relevant phenotypes (In vivo evolution)	443 longitudinal isolates from 39 young cystic fibrosis patients over 10 years	Identification of phenotypic changes that deviate from expected evolutionary trajectories	*mexZ, nfxB, nalDmucA, algU, retS/gacAS/rsmA gyrA and gyrB47*	Drug efflux pumps, mucoidity regulators, ciprofloxacin resistance	[307]

Abbreviations: AMR, antimicrobial resistance; CF, cystic fibrosis; CIP, ciprofloxacin; QS, quorum sensing; LPS, lipopolysaccharide; PA, *P. aeruginosa*; TCA, tricarboxylic acid cycle; T3SS, Type 3 secretion system; wgMLST, whole genome multi locus sequence typing.
